# Effect of Lifestyle on Asthma Control in Japanese Patients: Importance of Periodical Exercise and Raw Vegetable Diet

**DOI:** 10.1371/journal.pone.0068290

**Published:** 2013-07-09

**Authors:** Motoyasu Iikura, Siyan Yi, Yasunori Ichimura, Ai Hori, Shinyu Izumi, Haruhito Sugiyama, Koichiro Kudo, Tetsuya Mizoue, Nobuyuki Kobayashi

**Affiliations:** 1 Department of Respiratory Medicine, National Center for Global Health and Medicine, Tokyo, Japan; 2 Department of Epidemiology and International Health, National Center for Global Health and Medicine, Tokyo, Japan; National Jewish Health, United States of America

## Abstract

**Background:**

The avoidance of inhaled allergens or tobacco smoke has been known to have favorable effects on asthma control. However, it remains unclear whether other lifestyle-related factors are also related to asthma control. Therefore, a comprehensive study to examine the associations between various lifestyle factors and asthma control was conducted in Japanese asthmatic patients.

**Methods:**

The study subjects included 437 stable asthmatic patients recruited from our outpatient clinic over a one-year period. A written, informed consent was obtained from each participant. Asthma control was assessed using the asthma control test (ACT), and a structured questionnaire was administered to obtain information regarding lifestyle factors, including tobacco smoking, alcohol drinking, physical exercise, and diet. Both bivariate and multivariate analyses were conducted.

**Results:**

The proportions of total control (ACT = 25), well controlled (ACT = 20-24), and poorly controlled (ACT < 20) were 27.5%, 48.1%, and 24.5%, respectively. The proportions of patients in the asthma treatment steps as measured by Global Initiative for Asthma 2007 in step 1, step 2, step 3, step 4, and step 5 were 5.5%, 17.4%, 7.6%, 60.2%, and 9.4%, respectively. Body mass index, direct tobacco smoking status and alcohol drinking were not associated with asthma control. On the other hand, younger age (< 65 years old), passive smoking, periodical exercise (> 3 metabolic equivalents-h/week), and raw vegetable intake (> 5 units/week) were significantly associated with good asthma control by bivariate analysis. Younger age, periodical exercise, and raw vegetable intake were significantly associated with good asthma control by multiple linear regression analysis.

**Conclusions:**

Periodical exercise and raw vegetable intake are associated with good asthma control in Japanese patients.

## Introduction

Bronchial asthma attacks are often observed in several situations, including allergen inhalation, smoking, alcohol drinking, exercise, and the use of non-steroidal anti-inflammatory drugs. To date, many investigators have reported relationships between several lifestyle factors and asthma incidence [1–7]. Increasing body mass index (BMI), passive smoking, and low income are risk factors for asthma incidence [1–4]. Daily intake of fresh fruit or vegetables in infancy decreases the risk of asthma occurrence [5]. Previous increased intakes of saturated fatty acids, myristic and palmitic acids, and butter appear to be related to the risk of current asthma in children [6]. More frequent consumption of fruit, vegetables, and fish was associated with a lower lifetime prevalence of asthma, whereas higher burger consumption was associated with higher lifetime asthma prevalence [8].

On the other hand, other studies have reported that there were no clear relationships between dietary patterns and asthma incidence [9,10]. These previous reports focused on the relationship between asthma incidence and past lifestyle factors including diet, but there have been few reports concerning the relationship between asthma control and daily lifestyle [11]. Moreover, inconsistent findings have been observed in the existing studies looking at factors associated with asthma control. The avoidance of inhaled allergens or tobacco smoke has been known to have favorable effects on asthma control. However, González Barcala and colleagues reported that alcohol drinking did not affect asthma control [12]. Similarly, Westermann and colleagues also did not find the relationship between asthma control and periodic exercise [13]. Moreover, it remains unclear whether other lifestyle-related factors are also related to asthma control. Therefore, a comprehensive study was conducted to examine the associations between various lifestyle factors and asthma control in Japanese asthmatic patients.

## Materials and Methods

### Ethics Statement

This study was approved by the Institutional Review Board of the National Center for Global Health and Medicine and a written informed consent was obtained from each participant. This study was conducted according to the principles expressed in the Declaration of Helsinki.

### Study Design

The study subjects included 437 stable asthmatic patients recruited from the outpatient clinic of the National Center for Global Health and Medicine, Tokyo, Japan in 2009–2010. Eligible patients were aged over 20 years and had a clinical diagnosis of asthma supported by one or more other characteristics: variability in peak expiratory flow of more than 20%; the airway reversibility by inhaled β2 agonist; hyperresponsiveness of methacholine challenge; recurrent dyspnea episode with wheezing. We excluded patients who could not fill in the questionnaire, or who did not visit the clinic regularly, or who was diagnosed as asthma within 3 months of the study entry.

Asthma control for the last four weeks was assessed using the asthma control test (ACT). A structured questionnaire was administered to obtain information regarding lifestyle factors, including tobacco smoking, alcohol drinking, physical exercise, dietary intakes, pets, living space, cleaning habits, occupation, medical expenses, and asthma diary record. The exercise was defined as the total amount of walking (2 metabolic equivalents (METs)), light exercise (2 METs), moderate exercise (4 METs), heavy exercise (6 METs), and gardening (2 METs). Concerning the dietary intakes, we collected information regarding the consumption of cooked vegetables, raw vegetables, citrus fruits, other fruits, vegetable and fruit mixed juice, vegetable juice, and 100% fruit juice. Raw vegetables referred uncooked, unprocessed vegetables, which are usually organic or wild vegetables. They include uncooked tomatoes, carrots and leafy greens. The amount of intakes was assessed by the conversion of “unit”, which was defined as the amount of food held on one hand.

### Statistical analyses

We assessed characteristics of participants and their bivariate association with asthma control levels using Pearson’s χ^2^ test or Fisher’s exact test for categorical variables and Student *t*-test, Mann–Whitney U test, or Kruskal-Wallis test for continuous variables. Additional analyses were conducted, stratified by sex (male and female) and age groups (≤ 64 years and > 64 years). A multiple linear regression model was then constructed to examine the association between asthma control scores and lifestyle-related factors. Two-sided *p*-values of < 0.05 were regarded as statistically significant. Data analyses were performed with STATA version 11.0 (Lakeway Drive College Station, TX, USA) or SPSS statics version 17.0.0 (IBM Japan, Tokyo, Japan).

**Figure 1:**
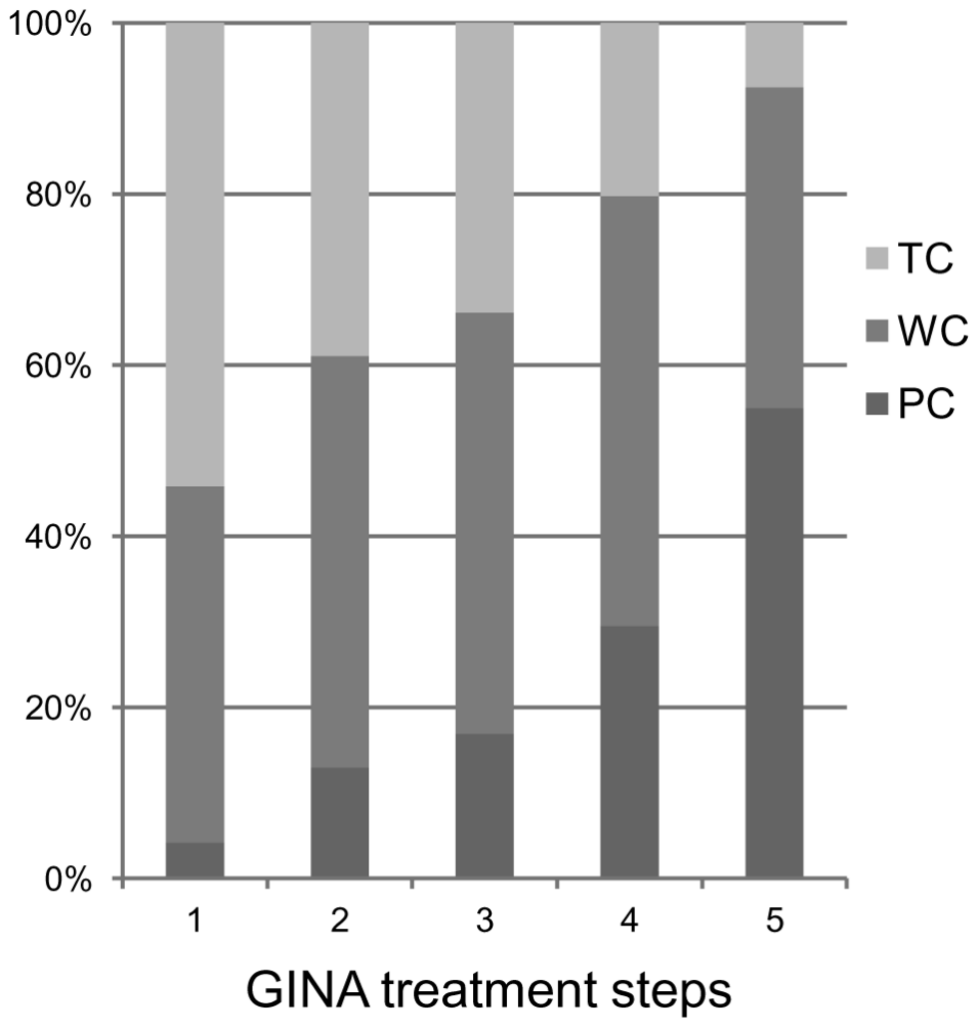
Asthma control status by GINA treatment steps (n=437). TC denotes total control; WC denotes well control; PC denotes poor control.

## Results

### Patients' characteristics

The patients' characteristics are shown in Table 1. The mean age of the patients was 64 years, and the average duration of asthma was 18 years. Sixty percent of the patients were atopic, 54.7% of patients were non-smokers, and current smokers accounted for only 6.6%. The comorbidities of the patients included allergic rhinitis (49.5%), allergic dermatitis (13.6%), sinusitis (29.0%), and chronic obstructive pulmonary disease (COPD) (11.0%). Regarding types of treatment they received, 93.2% of patients used inhaled corticosteroid (ICS), and 66.4% of patients used a long acting β2 agonist (LABA). The proportions of patients in the asthma treatment steps as measured by Global Initiative for Asthma (GINA) 2007 in step 1, step 2, step 3, step 4, and step 5 were 5.5%, 17.4%, 7.6%, 60.2%, and 9.4%, respectively. The proportion of patients with total control (ACT = 25), well control (ACT = 20-24), and poor control (ACT < 20) were 27.5%, 48.1%, and 24.5%, respectively (Table 1 Figure 1). Fifty-five percent of patients in step 5 were poorly controlled (Figure 1). Although the proportion of poorly controlled patients increased gradually depending on the enhanced treatment steps, a direct association between treatment steps and asthma control was not observed.

**Table 1:** Patients’ Characteristics (*n* = 437).

**Characteristics**		**Number (%)**
Male		204 (46.7)
Mean age (range)		64 y (51–74 y)
Mean age at the asthma onset (range)		46 y (26–59 y)
Atopic asthma		267 (61.1)
Smoking status	Non-smoker	260 (59.5)
	Ex-smoker	146 (33.4)
	Current smoker	31 (7.1)
Co-morbidity	Allergic rhinitis	216 (49.5)
	Sinusitis	127 (29.0)
	Hypertension	114 (26.0)
	Atopic dermatitis	60 (13.6)
	COPD	49 (11.3)
	Heart diseases	46 (10.5)
	DM	40 (9.1)
	AIA	27 (6.1)
	ABPA	11 (2.6)
	CSS	8 (1.8)
Drug treatment	ICS	407 (93.2)
	SFC (Adoair^®^)	138 (31.6)
	BUD (Pulmicort^®^)	134 (30.7)
	FP (Flutide^®^)	102 (23.3)
	HFA-BDP (Qvar^®^)	24 (5.5)
	CIC-BDP (Alvesco^®^)	9 (2.1)
	Systemic steroid	41 (9.4)
	LABA	290 (66.4)
	Oral β2 stimulant	7 (1.6)
	Stick β2 stimulant	27 (6.2)
	Theophylline	138 (31.6)
	LTRA	111 (25.4)
Control	Total control (ACT 25)	120 (27.5)
	Well control (ACT 20-24)	210 (48.1)
	Poor control (ACT <20)	107 (24.5)
GINA treatment step	1	24 (5.5)
	2	76 (17.4)
	3	33 (7.6)
	4	263 (60.2)
	5	41 (9.4)

DM: Diabetes mellitus; AIA: Aspirin-induced asthma; ABPA: Allergic bronchopulmonary aspergillosis; CSS: Churg–Strauss syndrome; ICS: inhaled corticosteroid; SFC: salmeterol/fluticasone propionate combination; BUD: budesonide; FP: fluticasone propionate; HFA: hydrofluoroalkane; BDP: beclomethasone dipropionate; CIC: ciclesonide; LTRA: leukotriene receptor antagonist

### Relationships between asthma control and smoking, drinking, and exercise

Table 2 shows the comparisons of median ACT scores by sex, age groups, BMI categories, smoking status, alcohol drinking status, and exercise amounts. The median ACT score was significantly higher in patients aged of 64 years or younger than in patients aged over 65 years (Table 2). More than 60% of patients aged under 64 maintained an ACT score of 25 (total control) (data not shown). Median ACT score was not significantly different among patients in non-smokers, past smokers, and current smokers. However, median ACT score was significantly lower in passive smokers compared to that in non-passive-smokers (*p* = 0.03). However, passive smoking was excluded by stepwise selection under the multiple linear regression analysis (Table 3). The median ACT score was also not significantly different among alcohol drinkers and non-drinkers.

**Table 2:** Comparisons of median ACT scores by sex, age, BMI, smoking, alcohol drinking, and exercise.

			**Total (*n* = 437)**	
		**n (%)**	**Median (IQR)**	***p***
Sex	Male	204 (46.7)	23 (20-25)	0.66
	Female	233 (53.3)	23 (19-25)	
Age group	≤ 64 years	221 (50.6)	23 (21-25)	0.03*
	> 64 years	216 (49.4)	22 (18-24)	
Body mass index (kg/m^2^)	≤ 22	36 (8.2)	23 (19-25)	0.42
	22-24.9	309 (70.7)	23 (21-25)	
	≥ 25	92 (21.1)	22 (18-24)	
Smoking	Non smokers	279 (63.8)	23 (19-25)	0.65
	Past smokers	128 (29.3)	22 (20-24)	
	Current smokers	30 (6.9)	22 (20-25)	
Passive smoking	No	281 (64.3)	23 (20-25)	0.03*
	Yes	156 (35.7)	22 (19-24)	
Alcohol drinking	No	240 (54.9)	22 (19-25)	0.23
	Yes	197 (45.1)	23 (20-25)	
Total amount of exercise (minutes/week)	≤ 80 minutes	219 (50.1)	22 (19-25)	0.006*
	> 80 minutes	218 (49.9)	23 (21-25)	
Total amount of exercise (METs-h/week)	≤ 3 METs-h/week	222 (50.8)	22 (19-25)	0.005*
	> 3 METs-h/week	215 (49.2)	23 (21-25)	

* Mann–Whitney U test or Kruskal-Wallis test

METs-h metabolic equivalents-hourTotal exercise = walking + light exercise + moderate exercise + heavy exercise + gardening

Total exercise = walking + light exercise + moderate exercise + heavy exercise + gardening

Regarding exercise, the median ACT score was significantly higher among patients who exercised more than 80 minutes per week compared to that among patients who exercised 80 minutes per week or less (*p* = 0.006) (Table 2). In term of the amount of exercise, the median ACT score was significantly higher among patients who exercised more than 3 METs-h per week compared to that among patients who did 3 METs-h per week or less (*p* = 0.005). Multiple linear regression analysis confirmed the significance of the bivariate analysis (Table 3).

**Table 3:** Association between the ACT score and age, exercise, raw vegetable intake on multiple linear stepwise regression analysis.

	***β***	***S.E***	***t***	***p***
Age (< 65 yrs)	-0.146	0.364	3.134	0.002
Periodical exercise	0.152	0.371	-3.059	0.002
High raw vegetable intake	-0.096	0.364	-2.012	0.045

Periodical exercise indicates the amount of exercise more than 3 METs-h/week.

High raw vegetable intake indicates more than 5 units of raw vegetable intake per week.

R2 = 0.049, ANOVA p<0.001

### The relation of asthma control to diet

The comparisons of median ACT scores in levels of various vegetable and fruit intakes are shown in Table 4. The median ACT score was significantly higher among patients who consumed more than 5 units of raw vegetables per week compared to that among patients consuming five units or less of raw vegetables per week (*p* = 0.02). However, additional analyses stratified by gender and age groups showed that this association was found only in men (*p* = 0.001) and in patients aged > 64 years (*p* = 0.005) (Table 5 and Table 6). Similarly, as shown in Table 7, the median ACT score was significantly higher among patients who consumed > 1 unit of vegetable juice per week compared to that in patients consuming 1 unit or less of vegetable juice per week (*p* = 0.02), but only in patients aged 64 years or younger. In multiple linear regression analysis, raw fresh vegetable intake remained significantly associated with higher levels of asthma control (*p* = 0.005) (Table 8).

**Table 4:** Comparisons of median ACT scores by various vegetable and fruit intakes in all subjects.

			**Total (*n*= 437)**	
		***n***	**Median (IQR)**	***p***
Cooked vegetables	≤ 7 units/week	153 (57.9)	23 (21-25)	0.205
	> 7 units/week	184 (42.1)	22 (19-25)	
Raw vegetables	≤ 5 units/week	220 (50.3)	21 (20-25)	0.02*
	> 5 units/week	217 (49.7)	23 (21-25)	
Total vegetables^†^	≤ 21 units/week	238 (54.5)	23 (21-25)	0.57
	> 21 units/week	199 (45.5)	22 (20-25)	
Citrus fruits	≤ 3 units/week	238 (56.5)	23 (20-25)	0.33
	> 3 units/week	183 (43.5)	22 (20-25)	
Other fruits	≤ 3 units/week	229 (55.9)	23 (20-25)	0.61
	> 3 units/week	181 (44.1)	23 (19-25)	
Total fruit intake**^*‡*^**	≤ 7 units/week	230 (53.9)	23 (20-25)	0.82
	> 7 units/week	197 (46.1)	23 (19-25)	

* Mann–Whitney U test. One unit was defined as the amount of food held on one hand.

† Cooked vegetables (x 2) + raw vegetables.

‡ Citrus fruits + other fruits.

## Discussion

Several studies have previously reported the relationships between lifestyle factors and asthma incidence [1–7]. However, few reports have focused on the relationships between asthma control and lifestyle factors. A total of 437 asthmatic patients were interviewed in our outpatient clinic, and the relationships between asthma control and several lifestyle factors were investigated. The relationships of smoking or alcohol drinking with asthma have already been reported in several articles. Radon and colleagues reported that passive smoking was a risk factor for asthma occurrence [3], while Bakirtas reported that passive smoking and low income were risk factors for asthma incidence [4]. Similar results were observed in the present study; patients who were exposed to passive smoking or who could not pay any medical expenses for asthma treatment, had a tendency to poor asthma control (data partly shown). Regarding lifestyle-related factors, González reported that alcohol drinking did not affect asthma control [12]. Similar results were obtained in the present study.

**Table 5:** Comparisons of median ACT scores by various vegetable and fruit intakes stratified by sex.

		**Male (*n*=204)**		**Female (*n*= 233)**	
		**Median (IQR)**	p*	**Median (IQR)**	p*
Cooked vegetables	≤ 7 units/week	23 (20-25)	0.17	23 (20-25)	0.66
	> 7 units/week	22 (19-24)		23 (20-25)	
Raw vegetables	≤ 5 units/week	21 (19-24)	0.001*	23 (20-25)	0.88
	> 5 units/week	23 (21-25)		23 (19-25)	
Total vegetables**^*†*^**	≤ 21 units/week	23 (21-25)	0.79	23 (20-25)	0.60
	> 21 units/week	23 (20-25)		22 (19-25)	
Citrus fruits	≤ 3 units/week	23 (20-25)	0.41	23 (20-25)	0.66
	> 3 units/week	22.0 (20-25)		22 (19-25)	
Other fruits	≤ 3 units/week	23 (20-25)	0.62	22 (20-25)	0.81
	> 3 units/week	22 (19-25)		23 (19-25)	
Total fruit intake**^*‡*^**	≤ 7 units/week	23 (19-25)	0.19	23 (19-25)	0.67
	> 7 units/week	22 (20-25)		23 (19-24)	

* Mann–Whitney U test.

† Cooked vegetables (x 2) + raw vegetables.

‡ Citrus fruits + other fruits.

Lucas and colleagues insisted on the importance of physical activity on decreases in asthma prevalence [14]. On the other hand, Westermann found that there was no relationship between asthma control and periodic exercise [13]. However, moderate exercise (> 80 min/week) was found to be associated with good asthma control in the present study. The Japanese government has recommended that 4 METs-h/week exercise is required for the prevention of lifestyle-related diseases. In the present study, patients with more than 3 METs-h/week exercise had good asthma control.

**Table 6:** Comparisons of median ACT scores by various vegetable and fruit intakes stratified by age groups.

		**Age ≤ 64 years (*n* =221)**		**Age > 64 years (*n* = 216)**	
		**Median (IQR)**	p*	**Median (IQR)**	p*
Cooked vegetables	≤ 7 units/week	23 (21-25)	0.262	22 (18-25)	0.729
	> 7 units/week	23 (19-25)		22 (18-24)	
Raw vegetables	≤ 5 units/week	23 (21-25)	0.49	21 (17-24)	0.005*
	> 5 units/week	23 (21-25)		23 (20-25)	
Total vegetables**^*†*^**	≤ 21 units/week	23 (21-25)	0.60	22 (18-24)	0.99
	> 21 units/week	23 (21-25)		22 (18-24)	
Citrus fruits	≤ 3 units/week	23 (21-25)	0.39	22 (19-24)	0.85
	> 3 units/week	23 (20-25)		22 (18-25)	
Other fruits	≤ 3 units/week	23 (21-25)	0.85	22 (18-24)	0.77
	> 3 units/week	23 (21-25)		22 (19-24)	
Total fruit intake**^*‡*^**	≤ 7 units/week	23 (21-25)	0.58	23 (19-25)	0.63
	> 7 units/week	23 (19-25)		22 (19-24)	

* Mann–Whitney U test.

† Cooked vegetables (x 2) + raw vegetables.

‡ Citrus fruits + other fruits.

Several empirical studies have investigated the effects of dietary intakes on asthma. Frode reported that daily intakes of fresh fruit or vegetables in infancy decreased the risk of asthma in school-age children [5]. Rodriguez found that increased intakes of saturated fatty acids, myristic and palmitic acids, and butter appeared to be related to the risk of current asthma in children [6]. Other reports mentioned that intakes of α-linolenic acid and a low ratio of n-6:n-3 PUFA were associated with decreased exhaled NO and improved asthma control [8]. Nagel reported that more frequent consumption of fruit, vegetables, and fish was associated with a lower lifetime prevalence of asthma, whereas high burger consumption was associated with higher lifetime asthma prevalence [9]. On the other hand, other investigators reported that there were no clear relationships between dietary patterns and asthma incidence [10,11].

**Table 7:** Comparisons of median ACT scores by various vegetable and fruit intakes stratified by age groups.

		Age ≤ 64 years (*n* =221)		Age > 64 years (*n* = 216)	
		Median (IQR)	*p* *	Median (IQR)	*p* *
Vegetable and fruit mixed juice	≤ 1 unit/ week	23 (21-25)	0.63	22 (18-24)	0.63
	> 1 unit/ week	22 (20-25)		22 (18-24)	
Vegetable juice	≤ 1 unit/ week	23 (20-25)	0.02*	22 (18-25)	0.65
	> 1 unit/ week	24 (21-25)		22 (19-24)	
100% fruit juice	≤ 3 units/week	23 (21-25)	0.70	22 (18-24)	0.95
	> 3 units/week	23 (21-25)		22 (20-24)	
Total juice intake^§^	≤ 1 unit/ week	23 (21-25)	0.71	22 (18-24)	0.87
	> 1 unit/ week	23 (20-25)		23 (19-24)	

* Mann–Whitney U test.

§ Vegetable and fruit mixed juice + vegetable juice + 100% fruit juice.

These previous reports focused on the relationships between asthma incidence and diet, while the present study examined the relationships between asthma control and diet. Particularly fresh vegetable, but not heated vegetable, intakes were associated with good asthma control in the present study. The possible explanations for this relationship remain to be investigated. In general, flavonoids and related polyphenolic compounds in vegetables are lost with heating. There is a report that flavonoids and related polyphenolic compounds had significant anti-inflammatory activity [15]. Recently, Wood reported the importance of intakes of antioxidants in vegetables for asthma [16]. Further studies are required to elucidate the relationship between flavonoids or antioxidants and asthma control.

**Table 8:** Association between vegetable or fruit intake and the ACT score on multiple linear regression.

	***β***	***S.E***	***t***	***p***
Cooked vegetables	-0.051	0.027	-1.027	0.31
Raw vegetables	0.133	0.031	2.794	0.005*
Citrus fruits	0.025	0.033	0.495	0.62
Other fruits	0.004	0.036	0.092	0.93
Mixed juice	-0.032	0.063	-0.671	0.50
Vegetable juice	0.036	0.063	0.763	0.45
100% fruit juice	-0.027	0.084	-0.568	0.57

Multiple linear regression adjusted for sex (male/female), age (continuous), exercise (METs-h/week), smoking status (yes/no), passive smoking status (yes/no), alcohol drinking (yes/no), and GINA step (categorical).

In general, citrus fruits contain more amount of vitamin C than other fruits. Previous reports indicated the relationship between consumption of citrus fruits and incidence of asthma [17,18]. Furthermore, citrus fruits contain anti-inflammatory effect [19]. However, we could not find the relation between the consumption of citrus fruits and asthma control in our study. Although citrus fruits are also included in fruit mixed juice and 100% fruit juice, the relation between asthma control and fruit mixed juice or 100% fruit juice was not observed. One of the possible reasons is the genotype of the patient because citrus fruits may influence the sensitivity of the treatment of asthma [20].

Findings from this study are strengthened by the use of reliable and standardized questionnaire to measure asthma control levels. Diez reported the relationships between asthma control and several risk factors, including sex, race, BMI, smoking, level of education, and habitual activity, in Spanish asthmatic patients [21]. They used the asthma control questionnaire (ACQ) to evaluate asthma control. This questionnaire reflected asthma control for the most recent week. In the present study, we used the ACT questionnaire, which reflects longer term (recent one month) of asthma control than the ACQ. For this reason, we believe that the ACT is better than the ACQ for evaluation of asthma control when comparing lifestyle factors.

The statistical significance of the relation between asthma control and exercise or raw vegetable diet intake was observed in our multiple linear regression analysis. However, the adjusted R squared was 0.049, indicating that the correlation coefficient was relatively weak. Interpretation of the results of our study should be made with caution. Since this study was conducted by only one institution, further multicenter studies are required for universalization of our results.

In conclusion, periodical exercise and raw vegetable intakes are associated with good asthma control in Japanese patients.
